# Editorial: Regulatory Mechanisms of Early Intracellular Signaling in T Lymphocytes

**DOI:** 10.3389/fcell.2021.676949

**Published:** 2021-04-09

**Authors:** Enrique Aguado, Ewoud B. Compeer, Arkadiusz Miazek

**Affiliations:** ^1^Institute of Biomedical Research Cadiz (INIBICA), Cádiz, Spain; ^2^Department of Biomedicine, Biotechnology and Public Health (Immunology), University of Cádiz, Cádiz, Spain; ^3^Kennedy Institute of Rheumatology, Nuffield Department of Orthopaedics, Rheumatology and Musculoskeletal Sciences, University of Oxford, Oxford, United Kingdom; ^4^Department of Biochemistry and Molecular Biology, Wroclaw University of Environmental and Life Sciences, Wroclaw, Poland

**Keywords:** TCR, tyrosine kinases, tyrosine phospatases, transmembrane adaptor proteins, bioenergetics, immune synapse, membrane dynamics

Activation of T lymphocytes constitutes a central event in adaptive immune responses. Antigen recognition by T cells through the T cell receptor (TCR) triggers a cascade of intracellular signals leading to T cell activation and development of effector functions. Proper TCR signaling requires sequential activities of Lck and ZAP-70 tyrosine kinases, resulting in phosphorylation of tyrosine residues located in the CD3 ITAMs and adaptor protein LAT, respectively (Courtney et al., 2018). Although there has been a huge increase in knowledge on activatory intracellular signaling in recent years, much less is known about the molecular mechanisms negatively regulating early TCR intracellular signals.

This Research Topic contains 9 articles covering different aspects in the field of intracellular signaling coupled to the TCR/CD3 complex. T lymphocyte activation is initiated by interaction of TCRs with agonistic peptide-major histocompatibility complexes (pMHC) on the surface of antigen presenting cells (APCs). This triggers dramatic remodeling and reorganization of the T cell's 3D endocytic network to efficiently deliver TCR and TCR signaling proteins into a highly-organized 2D interface where signaling is integrated called the immunological synapse (IS) (Dustin and Choudhuri, 2016). Mastrogiovanni et al. comprehensively reviews the crucial role T cells' cytoskeleton plays in T cell activation by aiding the formation of the immunological synapse, regulating antigen recognition, and delivery of crucial signaling proteins into the IS.

One example of such crucial family of proteins are the co-receptors, which strengthen T-cell responses by many orders of magnitude. Morch et al. reviews three possible mechanisms explaining how co-receptors so profoundly amplify TCR signaling: (i) the Lck recruitment model, (ii) the pseudodimer model, and (iii) the two-step coreceptor recruitment to partially triggered TCRs model.

Felce et al. provides new data illustrating that other molecules recruited to the immunological synapse can contribute to T cell activation as well. More specifically, using a knock-out screen they identify various G-protein coupled receptors that contribute to T cell activation. Mutating G-protein coupled receptor CXCR4, for example, perturbs its recruitment into the immunological synapse and abolishes its contribution to activation of primary human CD4 T cells.

In parallel, Rudd summarizes the history of events leading to the development of the tyrosine kinase-mediated “TCR signaling paradigm” in T cells and discusses its importance for the design of new therapeutic approaches.

Building on this knowledge Castro-Sanchez et al. reviews the role of tyrosine phosphatases -that remove phosphates from tyrosines- as crucial regulators of T cells activation, and their potential as therapeutic agents in autoimmune disorders and cancer. More specifically, this collection includes new data about the specific regulatory role of phosphorylation of LAT tyrosine residue 132 Arbulo-Echevarria et al.. It had previously been shown that the phosphorylation of tyrosine 132 of human LAT form has slower phosphorylation kinetics than the other functionally relevant tyrosines, due to the presence of a glycine residue preceding the tyrosine (Gly131) (Houtman et al., 2005; Lo et al., 2019). Now, we confirm that a LAT mutant in which glycine 131 has been substituted by an aspartate (LATG131D) increases TCR signaling, and also that T cells expressing the LATG131D mutant are more sensitive to inhibition of IL-2 production by pre-treatment with anti-CD3, which points to a possible role of this residue in the generation of anergy.

Two other reviews in this collection discuss how the TCR organization at the IS affects signaling. Balagopalan et al. discusses organization of microclusters, as well as the kinetics of recruitment and disassociation of molecules from microclusters in T cells. In their article, authors explain in detail the kinetics of recruitment and segregation of molecules from microclusters, and the role of post-translational modifications in the downregulation of the microcluster-associated signaling molecules. In parallel (Bunnell et al., 2002; Lee et al., 2002). Pathan-Chhatbar et al. focuses on the role of the interaction between plasma membrane cholesterol and the TCR clustering. The authors describe the opposing roles that this interaction may have in the context of T cell activation, discussing their own recently published data as well as those of other groups. In their review, Schamel and collaborators propose an interesting model by which cholesterol keeps in check TCRs that have not bound an antigenic pMHC, but at the same time is able to favor the formation of nanoclusters that increase TCR avidity, and thus their “activatability.”

Finally, this collection includes a review by Castellano and Molinier-Frenkel on the role of amino acid catabolizing enzymes (IDO1/2, TDO, Arg1/2 and IL4I1) as regulators of T cell activation and differentiation Castellano and Molinier-Frenkel.

In summary, this Research Topic brings together a number of interesting contributions, focused on the mechanisms by which the TCR/CD3 complex transduces intracellular signals, and some of the mechanisms that regulate them. Technical advances are allowing to deepen into the role of tyrosine kinases and phosphatases as regulators of TCR signaling, the function of costimulatory and coinhibitory receptors, the relevance of bioenergetics for T cell activation, and the importance of membrane dynamics and nanoscale organization of TCR associated molecules in the regulation of TCR signaling.

## Author Contributions

EA wrote the first draft of the manuscript and updated the last version. EBC and AM corrected and completed the initial draft. All authors contributed to the article and approved the submitted version.

## Conflict of Interest

The authors declare that the research was conducted in the absence of any commercial or financial relationships that could be construed as a potential conflict of interest.
